# Supplementary material for: Pyruvate Dehydrogenase Kinase 1 inhibition mediated oxidative phosphorylation enhancement in cartilage promotes osteoarthritis progression
Source: BMC Musculoskelet Disord. 2023 Jul 20;24:597. doi: 10.1186/s12891-023-06585-6 (PMC10357736; doi:10.1186/s12891-023-06585-6)
Supplement: Supplementary file 1 — Additional file 1. [file 12891_2023_6585_MOESM1_ESM.zip › KEGG.Gsea.1653622432464/KEGG_HEMATOPOIETIC_CELL_LINEAGE.html]

Details for gene set KEGG\_HEMATOPOIETIC\_CELL\_LINEAGE[GSEA]

|  || Dataset | 55457\_collapsed\_to\_symbols.cls.cls #OA\_versus\_normal.cls.cls #OA\_versus\_normal\_repos |
| Phenotype | cls.cls#OA\_versus\_normal\_repos |
| Upregulated in class | normal |
| GeneSet | KEGG\_HEMATOPOIETIC\_CELL\_LINEAGE |
| Enrichment Score (ES) | -0.18382975 |
| Normalized Enrichment Score (NES) | -0.77419084 |
| Nominal p-value | 0.8939641 |
| FDR q-value | 0.9769286 |
| FWER p-Value | 1.0 |
Table: GSEA Results Summary

  

Fig 1: Enrichment plot: KEGG\_HEMATOPOIETIC\_CELL\_LINEAGE      
 Profile of the Running ES Score & Positions of GeneSet Members on the Rank Ordered List

  

| SYMBOL | TITLE | RANK IN GENE LIST | RANK METRIC SCORE | RUNNING ES | CORE ENRICHMENT || 1 | IL11RA | interleukin 11 receptor subunit alpha [Source:HGNC Symbol;Acc:HGNC:5967] | 500 | 0.659 | -0.0119 | Yes |
| 2 | FCGR1A | Fc fragment of IgG receptor Ia [Source:HGNC Symbol;Acc:HGNC:3613] | 633 | 0.610 | 0.0030 | Yes |
| 3 | CSF1R | colony stimulating factor 1 receptor [Source:HGNC Symbol;Acc:HGNC:2433] | 713 | 0.590 | 0.0211 | Yes |
| 4 | FLT3LG | fms related receptor tyrosine kinase 3 ligand [Source:HGNC Symbol;Acc:HGNC:3766] | 894 | 0.544 | 0.0295 | Yes |
| 5 | CD2 | CD2 molecule [Source:HGNC Symbol;Acc:HGNC:1639] | 1306 | 0.457 | 0.0162 | Yes |
| 6 | CD55 | CD55 molecule (Cromer blood group) [Source:HGNC Symbol;Acc:HGNC:2665] | 1619 | 0.411 | 0.0088 | Yes |
| 7 | MS4A1 | membrane spanning 4-domains A1 [Source:HGNC Symbol;Acc:HGNC:7315] | 1652 | 0.406 | 0.0230 | Yes |
| 8 | CD9 | CD9 molecule [Source:HGNC Symbol;Acc:HGNC:1709] | 1752 | 0.392 | 0.0314 | Yes |
| 9 | GP9 | glycoprotein IX platelet [Source:HGNC Symbol;Acc:HGNC:4444] | 1826 | 0.381 | 0.0414 | Yes |
| 10 | ITGA4 | integrin subunit alpha 4 [Source:HGNC Symbol;Acc:HGNC:6140] | 1854 | 0.379 | 0.0549 | Yes |
| 11 | CD33 | CD33 molecule [Source:HGNC Symbol;Acc:HGNC:1659] | 1909 | 0.372 | 0.0660 | Yes |
| 12 | CD14 | CD14 molecule [Source:HGNC Symbol;Acc:HGNC:1628] | 1928 | 0.369 | 0.0798 | Yes |
| 13 | CD1E | CD1e molecule [Source:HGNC Symbol;Acc:HGNC:1638] | 2038 | 0.357 | 0.0860 | Yes |
| 14 | TNF | tumor necrosis factor [Source:HGNC Symbol;Acc:HGNC:11892] | 2098 | 0.350 | 0.0958 | Yes |
| 15 | KIT | "KIT proto-oncogene, receptor tyrosine kinase [Source:HGNC Symbol;Acc:HGNC:6342]" | 2117 | 0.348 | 0.1087 | Yes |
| 16 | IL7 | interleukin 7 [Source:HGNC Symbol;Acc:HGNC:6023] | 2320 | 0.324 | 0.1063 | Yes |
| 17 | EPO | erythropoietin [Source:HGNC Symbol;Acc:HGNC:3415] | 2369 | 0.318 | 0.1157 | Yes |
| 18 | CD38 | CD38 molecule [Source:HGNC Symbol;Acc:HGNC:1667] | 2416 | 0.312 | 0.1250 | Yes |
| 19 | ITGA1 | integrin subunit alpha 1 [Source:HGNC Symbol;Acc:HGNC:6134] | 2421 | 0.311 | 0.1375 | Yes |
| 20 | CD37 | CD37 molecule [Source:HGNC Symbol;Acc:HGNC:1666] | 2451 | 0.308 | 0.1479 | Yes |
| 21 | CD59 | CD59 molecule (CD59 blood group) [Source:HGNC Symbol;Acc:HGNC:1689] | 2500 | 0.303 | 0.1566 | Yes |
| 22 | CR2 | complement C3d receptor 2 [Source:HGNC Symbol;Acc:HGNC:2336] | 2584 | 0.292 | 0.1622 | Yes |
| 23 | CD1B | CD1b molecule [Source:HGNC Symbol;Acc:HGNC:1635] | 2697 | 0.279 | 0.1650 | Yes |
| 24 | CD19 | CD19 molecule [Source:HGNC Symbol;Acc:HGNC:1633] | 2757 | 0.274 | 0.1717 | Yes |
| 25 | CD3D | CD3d molecule [Source:HGNC Symbol;Acc:HGNC:1673] | 2771 | 0.273 | 0.1819 | Yes |
| 26 | CD3E | CD3e molecule [Source:HGNC Symbol;Acc:HGNC:1674] | 2919 | 0.259 | 0.1811 | No |
| 27 | CD5 | CD5 molecule [Source:HGNC Symbol;Acc:HGNC:1685] | 3054 | 0.245 | 0.1807 | No |
| 28 | CD1C | CD1c molecule [Source:HGNC Symbol;Acc:HGNC:1636] | 3354 | 0.216 | 0.1663 | No |
| 29 | CD1D | CD1d molecule [Source:HGNC Symbol;Acc:HGNC:1637] | 3516 | 0.201 | 0.1620 | No |
| 30 | CSF2 | colony stimulating factor 2 [Source:HGNC Symbol;Acc:HGNC:2434] | 3699 | 0.185 | 0.1554 | No |
| 31 | FCER2 | Fc fragment of IgE receptor II [Source:HGNC Symbol;Acc:HGNC:3612] | 3785 | 0.178 | 0.1561 | No |
| 32 | CSF2RA | colony stimulating factor 2 receptor subunit alpha [Source:HGNC Symbol;Acc:HGNC:2435] | 3797 | 0.178 | 0.1626 | No |
| 33 | IL9R | interleukin 9 receptor [Source:HGNC Symbol;Acc:HGNC:6030] | 4118 | 0.144 | 0.1435 | No |
| 34 | CD8B | CD8b molecule [Source:HGNC Symbol;Acc:HGNC:1707] | 4226 | 0.135 | 0.1408 | No |
| 35 | IL2RA | interleukin 2 receptor subunit alpha [Source:HGNC Symbol;Acc:HGNC:6008] | 4292 | 0.129 | 0.1410 | No |
| 36 | CD22 | CD22 molecule [Source:HGNC Symbol;Acc:HGNC:1643] | 4296 | 0.128 | 0.1460 | No |
| 37 | IL11 | interleukin 11 [Source:HGNC Symbol;Acc:HGNC:5966] | 4327 | 0.126 | 0.1489 | No |
| 38 | CD34 | CD34 molecule [Source:HGNC Symbol;Acc:HGNC:1662] | 4341 | 0.125 | 0.1530 | No |
| 39 | CD4 | CD4 molecule [Source:HGNC Symbol;Acc:HGNC:1678] | 4465 | 0.116 | 0.1482 | No |
| 40 | EPOR | erythropoietin receptor [Source:HGNC Symbol;Acc:HGNC:3416] | 4596 | 0.105 | 0.1423 | No |
| 41 | FLT3 | fms related receptor tyrosine kinase 3 [Source:HGNC Symbol;Acc:HGNC:3765] | 4657 | 0.099 | 0.1418 | No |
| 42 | DNTT | DNA nucleotidylexotransferase [Source:HGNC Symbol;Acc:HGNC:2983] | 4792 | 0.088 | 0.1349 | No |
| 43 | CSF3 | colony stimulating factor 3 [Source:HGNC Symbol;Acc:HGNC:2438] | 4995 | 0.073 | 0.1222 | No |
| 44 | CD1A | CD1a molecule [Source:HGNC Symbol;Acc:HGNC:1634] | 5142 | 0.060 | 0.1133 | No |
| 45 | IL5 | interleukin 5 [Source:HGNC Symbol;Acc:HGNC:6016] | 5277 | 0.050 | 0.1049 | No |
| 46 | THPO | thrombopoietin [Source:HGNC Symbol;Acc:HGNC:11795] | 5365 | 0.043 | 0.0998 | No |
| 47 | GP1BB | glycoprotein Ib platelet subunit beta [Source:HGNC Symbol;Acc:HGNC:4440] | 5457 | 0.037 | 0.0943 | No |
| 48 | GYPA | glycophorin A (MNS blood group) [Source:HGNC Symbol;Acc:HGNC:4702] | 5672 | 0.018 | 0.0783 | No |
| 49 | ITGAM | integrin subunit alpha M [Source:HGNC Symbol;Acc:HGNC:6149] | 5820 | 0.007 | 0.0671 | No |
| 50 | TPO | thyroid peroxidase [Source:HGNC Symbol;Acc:HGNC:12015] | 6467 | -0.040 | 0.0184 | No |
| 51 | HLA-DRA | "major histocompatibility complex, class II, DR alpha [Source:HGNC Symbol;Acc:HGNC:4947]" | 6550 | -0.046 | 0.0138 | No |
| 52 | CD8A | CD8a molecule [Source:HGNC Symbol;Acc:HGNC:1706] | 6579 | -0.048 | 0.0136 | No |
| 53 | CD7 | CD7 molecule [Source:HGNC Symbol;Acc:HGNC:1695] | 6622 | -0.051 | 0.0125 | No |
| 54 | IL4 | interleukin 4 [Source:HGNC Symbol;Acc:HGNC:6014] | 6673 | -0.054 | 0.0108 | No |
| 55 | CD3G | CD3g molecule [Source:HGNC Symbol;Acc:HGNC:1675] | 6961 | -0.077 | -0.0084 | No |
| 56 | GP1BA | glycoprotein Ib platelet subunit alpha [Source:HGNC Symbol;Acc:HGNC:4439] | 6991 | -0.079 | -0.0074 | No |
| 57 | HLA-DRB1 | "major histocompatibility complex, class II, DR beta 1 [Source:HGNC Symbol;Acc:HGNC:4948]" | 7087 | -0.086 | -0.0113 | No |
| 58 | IL1A | interleukin 1 alpha [Source:HGNC Symbol;Acc:HGNC:5991] | 7842 | -0.144 | -0.0642 | No |
| 59 | ITGA2 | integrin subunit alpha 2 [Source:HGNC Symbol;Acc:HGNC:6137] | 7857 | -0.145 | -0.0593 | No |
| 60 | CD36 | CD36 molecule [Source:HGNC Symbol;Acc:HGNC:1663] | 7985 | -0.154 | -0.0629 | No |
| 61 | GP5 | glycoprotein V platelet [Source:HGNC Symbol;Acc:HGNC:4443] | 8215 | -0.173 | -0.0737 | No |
| 62 | ITGA6 | integrin subunit alpha 6 [Source:HGNC Symbol;Acc:HGNC:6142] | 9112 | -0.240 | -0.1337 | No |
| 63 | IL5RA | interleukin 5 receptor subunit alpha [Source:HGNC Symbol;Acc:HGNC:6017] | 9204 | -0.248 | -0.1305 | No |
| 64 | IL7R | interleukin 7 receptor [Source:HGNC Symbol;Acc:HGNC:6024] | 9233 | -0.251 | -0.1224 | No |
| 65 | CSF3R | colony stimulating factor 3 receptor [Source:HGNC Symbol;Acc:HGNC:2439] | 9247 | -0.252 | -0.1130 | No |
| 66 | MME | membrane metalloendopeptidase [Source:HGNC Symbol;Acc:HGNC:7154] | 9287 | -0.255 | -0.1056 | No |
| 67 | CSF1 | colony stimulating factor 1 [Source:HGNC Symbol;Acc:HGNC:2432] | 9613 | -0.284 | -0.1193 | No |
| 68 | IL3 | interleukin 3 [Source:HGNC Symbol;Acc:HGNC:6011] | 9850 | -0.306 | -0.1251 | No |
| 69 | TFRC | transferrin receptor [Source:HGNC Symbol;Acc:HGNC:11763] | 10184 | -0.339 | -0.1371 | No |
| 70 | IL1R2 | interleukin 1 receptor type 2 [Source:HGNC Symbol;Acc:HGNC:5994] | 10262 | -0.347 | -0.1288 | No |
| 71 | ITGB3 | integrin subunit beta 3 [Source:HGNC Symbol;Acc:HGNC:6156] | 10310 | -0.352 | -0.1180 | No |
| 72 | IL4R | interleukin 4 receptor [Source:HGNC Symbol;Acc:HGNC:6015] | 10902 | -0.417 | -0.1469 | No |
| 73 | CR1 | complement C3b/C4b receptor 1 (Knops blood group) [Source:HGNC Symbol;Acc:HGNC:2334] | 11376 | -0.483 | -0.1639 | No |
| 74 | ANPEP | "alanyl aminopeptidase, membrane [Source:HGNC Symbol;Acc:HGNC:500]" | 11404 | -0.487 | -0.1460 | No |
| 75 | CD44 | CD44 molecule (Indian blood group) [Source:HGNC Symbol;Acc:HGNC:1681] | 11439 | -0.494 | -0.1283 | No |
| 76 | KITLG | KIT ligand [Source:HGNC Symbol;Acc:HGNC:6343] | 11516 | -0.503 | -0.1135 | No |
| 77 | IL6R | interleukin 6 receptor [Source:HGNC Symbol;Acc:HGNC:6019] | 11615 | -0.519 | -0.0998 | No |
| 78 | ITGA2B | integrin subunit alpha 2b [Source:HGNC Symbol;Acc:HGNC:6138] | 11645 | -0.524 | -0.0805 | No |
| 79 | IL1B | interleukin 1 beta [Source:HGNC Symbol;Acc:HGNC:5992] | 12105 | -0.622 | -0.0907 | No |
| 80 | IL3RA | interleukin 3 receptor subunit alpha [Source:HGNC Symbol;Acc:HGNC:6012] | 12380 | -0.703 | -0.0831 | No |
| 81 | IL1R1 | interleukin 1 receptor type 1 [Source:HGNC Symbol;Acc:HGNC:5993] | 12419 | -0.713 | -0.0567 | No |
| 82 | IL6 | interleukin 6 [Source:HGNC Symbol;Acc:HGNC:6018] | 12460 | -0.730 | -0.0297 | No |
| 83 | ITGA5 | integrin subunit alpha 5 [Source:HGNC Symbol;Acc:HGNC:6141] | 12474 | -0.737 | -0.0004 | No |
| 84 | HLA-DRB4 | "major histocompatibility complex, class II, DR beta 4 [Source:HGNC Symbol;Acc:HGNC:4952]" | 12636 | -0.815 | 0.0206 | No |
Table: GSEA details [plain text format]

  

Fig 2: KEGG\_HEMATOPOIETIC\_CELL\_LINEAGE      
 Blue-Pink O' Gram in the Space of the Analyzed GeneSet

  

Fig 3: KEGG\_HEMATOPOIETIC\_CELL\_LINEAGE: Random ES distribution      
 Gene set null distribution of ES for **KEGG\_HEMATOPOIETIC\_CELL\_LINEAGE**
